# Reproductive coercion as a form of family violence against immigrant and refugee women in Australia

**DOI:** 10.1371/journal.pone.0275809

**Published:** 2022-11-03

**Authors:** Mariyam Suha, Linda Murray, Deborah Warr, Jasmin Chen, Karen Block, Adele Murdolo, Regina Quiazon, Erin Davis, Cathy Vaughan

**Affiliations:** 1 School of Geography, Earth and Atmospheric Sciences, University of Melbourne, Melbourne, Australia; 2 School of Medicine, University of Tasmania, Hobart, Australia; 3 College of Health, Massey University, Wellington, New Zealand; 4 Centre for Health Equity, Melbourne School of Population and Global Health, The University of Melbourne, Melbourne, Australia; 5 Multicultural Centre for Women’s Health, Melbourne, Australia; University of South Florida, UNITED STATES

## Abstract

Reproductive coercion (RC), generally considered a form of intimate partner violence (IPV), refers to perpetrator behaviours and actions that are intended to interfere with and control the autonomous decision-making of a person regarding their reproductive health. To date there are few studies that document RC as experienced by immigrant and refugee women. In this article, we explore cases of RC as described by women who were part of a larger qualitative study investigating violence against immigrant and refugee women in southern Australia. The study aimed to identify the types of RC detailed in immigrant and refugee women’s narratives, and to illustrate the contexts in which these experiences occurred. Analysis followed Baxter and Jack’s (2008) case study methodology; whereby particular “cases” are used to describe a phenomenon in context. Thirteen women from seven countries described experiences that fit definitions of RC. The cases describe various types of RC including violence during pregnancy with the intent of causing miscarriage, forced abortion, contraception sabotage and forced pregnancy. As well as intimate partners, some women described multiple perpetrators being complicit in their experience of RC, especially in regard to controlling women’s access to, and interactions with health services. More information is needed about immigrant and refugee women’s experiences of RC, and how vulnerability to multi-perpetrator violence affects health service access. In particular knowledge about how multi-perpetrator RC can affect consent processes for women who already face barriers to health care requires attention. Further research is required to address knowledge gaps about appropriate prevention and advocacy work about RC in refugee and migrant communities, and what training is needed for professionals in the family violence sector, women’s health services, women’s organisations, multicultural and ethno-specific services.

## Introduction

Reproductive coercion (RC), also referred to as reproductive abuse [1], is generally considered a form of intimate partner violence (IPV), whereby the perpetrator engages in behaviours and actions intended to interfere with and control the autonomous decision-making of a person regarding their reproductive health [2–4]. RC constitutes one of many possible tactics of power and control used by an abusive partner within the context of an intimate relationship and can take place in the absence of any physical violence [2, 4, 5]. Although RC is commonly perceived as being committed against a woman by a male intimate partner, women may also be subject to RC by multiple perpetrators such as in-laws and other family members [6]. In addition, government policies and legislation that restrict women’s access to reproductive health services, and cause gendered inequity in the health system, can facilitate RC [7, 8].

Labelled and purposefully studied for the first time in 2010, there is a paucity of research on RC [9]. Whilst it has been identified as a distinct phenomenon, it is linked to sexual coercion and other forms of controlling behaviour intended to maintain power and control in an intimate relationship [10]. Tarzia and Hegarty [11] suggest that a continuing lack of conceptual and definitional clarity around RC and abuse is a barrier to the establishment of a robust evidence base. For this reason, global estimates of the prevalence of RC amongst populations are limited or non-existent. The prevalence of RC among the Australian population is also unknown [9]. One Australian cross-sectional study conducted amongst a general practice-attending population documented that some participants who took part in the study had experienced a partner attempting to force a pregnancy or interfere with the use of contraception [1, 12]. Other studies have demonstrated that for women who had experienced violence at the hands of a former or current intimate partner, RC occurred concurrently with other forms of intimate partner sexual violence such as sexual assault, sexual abuse and intimate partner-forced sexual activity [2, 13]. The intersection of these various forms of IPV has been likened to wielding a web of control over victims, leaving them with diminished or no capacity to assert autonomy and sexual and reproductive agency [4, 5, 13]. Tarzia and Hegarty [11] explain that intimate partner violence and sexual violence are the *mechanisms* through which RC is perpetrated, and how such forms of violence co-occur and intersect requires greater exploration.

Controlling behaviours, ranging from the sabotaging of contraceptive methods to directing the use of contraceptives, and the forced continuation or termination of a pregnancy are all considered acts of RC [2, 5, 13]. Sabotage of contraception may include removing or damaging a condom to render it ineffective as a means for pregnancy prevention, removal of a contraceptive patch, hiding or throwing away oral contraceptives, refusal to pay for birth control and not withdrawing at an appropriate time where this is the agreed upon method of birth control [2, 4, 5, 14]. Women who have experienced IPV have also disclosed a compromised ability to negotiate or discuss the use of condoms with their partners, because they fear retaliation in the form of physical violence and emotional abuse [13, 14]. The perception that not using condoms signifies sexual exclusivity, commitment and fidelity within a relationship may be used as a controlling tactic by a partner, which presents difficulties for women wishing to use them as a method for managing their fertility or to promote their sexual health [14].

Forcing or pressuring a woman either to terminate or carry a pregnancy to term manifests in multiple ways. It includes intimate partners and extended family exerting sustained and intense pressure upon a woman to become pregnant and/ or continue a pregnancy, despite or against a woman’s own wishes. This pressure may be accompanied by threats from a partner to leave, and physical violence if she refuses or is unable to comply with such demands [2, 13, 14]. Conversely, women may be subject to physical violence with the intention of triggering miscarriage, or be forced to terminate pregnancies against their will [14]. These and other forms of RC have adverse effects on women’s sexual, reproductive, mental, and maternal health and violate her human rights.

Common health impacts of RC and associated IPV among women include sexually transmitted infections and diseases, urinary tract infections, chronic pain, injury and gastrointestinal issues [4, 5, 15]. There is evidence that violent intimate partners are more likely to have multiple, concurrent sexual partners and to force sex on a regular partner, increasing the risks of contracting sexually transmitted infections and injuries [15]. Forced sex, coupled with a partner’s refusal to use contraception can result in a pregnancy that was not planned by the woman [2, 14, 15]. The distress of becoming pregnant in these circumstances is accompanied by the poor maternal health outcomes associated with unplanned pregnancy, including miscarriage, induced abortion, stillbirth and other pregnancy-related complications [5, 15]. Experiences of IPV and other forms of family violence while pregnant have also been found to increase the risk of developing or exacerbating existing mental health issues such as depression, posttraumatic stress and suicidality among women [9]. However, there is limited exploration of mental health issues that co-occur with RC, with only one study finding that RC may be a significant contributor to adverse mental health outcomes [9].

Some studies suggest that factors such as relationship status, age and ethnicity may be related to the likelihood a woman is subject to RC [2, 9]. For instance, young women between the ages of 18–20 years have been found to be at higher risk of experiencing RC regardless of whether other forms of IPV or family violence are present [16, 17]. Less is known about experiences of RC among women in other age groups. Clark and colleagues [18], in their study of RC and co-occurring IPV, found that women who have experiences of RC were more likely to be single, in a dating relationship or unsure of their relationship status than women in long-term relationships. Single women were also found to be more likely to report IPV in the same relationship where RC occurred [18]. Data from the US also indicates a higher prevalence of RC among African American women, with significant links found between race/ethnicity and RC occurring as part of a complex sociocultural set of risk factors [9, 19, 20].

While RC affects women of all ages and backgrounds [6, 19], this paper specifically focuses on experiences of RC and its impact on immigrant and refugee women settled in Australia. Refugee women are at risk of experiencing sexual violence during their migration/refugee journey, resulting in reproductive consequences such as unplanned or unwanted pregnancy [21]. Migrant women experience gendered and racial discrimination within the Australian health system, which limits their access to reproductive health information, appropriate interpreting and timely healthcare [22, 23]. Australian immigration and health policies restrict access to reproductive health services to migrant women on temporary visas [24]. However, to date there are few studies that document RC as experienced by immigrant and refugee women. In this article, we explore cases of RC as described by participants who were part of a larger qualitative study investigating violence against immigrant and refugee women. The aim of this article was to identify the types of RC detailed in immigrant and refugee women’s narratives, and to illustrate the contexts in which these experiences occurred.

## Context and methodology: The ASPIRE project

This paper draws upon data from the ASPIRE project (Analysing Safety and Place in Immigrant and Refugee Experience), which investigated violence against immigrant and refugee women in the southern Australian states of Victoria and Tasmania. The ASPIRE project used a participatory approach, where university-based researchers collaborated with immigrant women’s health organisations, community-based organisations, and immigrant and refugee community members who co-design the research methodology and were involved in data collection and analysis processes [25]. The ASPIRE project incorporated feminist principles, including an understanding that the concerns, voices and lived experiences of women should be prioritized, and was framed by the understanding that violence against women is embedded in gendered inequality. However, in seeking to understand the specific experiences of immigrant and refugee women, gender alone was considered an insufficient lens for analysing women’s experiences of violence across different socioeconomic, ethno-cultural, trans-national, generational, geographic, political or other circumstances. Therefore, an intersectional approach was adopted [26, 27], which gave specific attention to the ways in which immigrant and refugee women’s experiences are shaped by the confluence of individual, social, institutional and immigration circumstances.

### Data collection

In-depth interviews were conducted with immigrant and refugee women who had experienced violence and were currently living in one of ASPIRE’s eight research sites across inner-city, outer metropolitan and regional settings in Victoria and Tasmania. Recruitment of women who had experienced violence was undertaken with the assistance of community-based organisations in these localities. A total of 46 in-depth interviews (33 in Victoria and 13 in Tasmania) were conducted with migrant and refugee women who had experienced IPV and other forms of family violence. Participants were provided with the option to be interviewed in their preferred language (their mother tongue, English or a third language), with professional female interpreters if required. Interviews were conducted by the authors or trained bilingual health educators. The interviews explored the dynamics and contexts of women’s experiences of violence, help-seeking, and use and knowledge of services.

### Data analysis

The initial analysis of the ASPIRE data utilized a deductive process, based on concepts drawn from the literature on violence against migrant and refugee women [25, 28], and an intersectional feminist theoretical framework. Participatory analysis workshops with bilingual interviewers supported the development of data coding frameworks. The coding framework was then refined using inductive coding to support identification of additional themes in the data.

Whilst the question guide did not specifically focus on RC, experiences of violence that fit the definition of RC frequently occurred. The analysis presented here adopted a qualitative case study methodology [29, 30] to investigate in-depth the dynamics of RC as experienced by participants. Here, “qualitative case study” refers to a form of qualitative research that draws upon multiple specific qualitative cases [31] to analyse complex phenomena within their bounded social contexts [32, 33]. In this analysis the case “unit” which guided data coding was the type of RC (forced abortion, violence during pregnancy with the intent to end the pregnancy, forced pregnancy, and sabotage of safe sexual practice), as described by one or multiple participants. As case study methodology is a qualitative, constructivist approach, results are not intended to be generalisable, but rather to elucidate the contexts in which RC occurred for immigrant and refugee women who participated in the ASPIRE study. Because of the dearth of research on RC in Australia, the advantage of employing a qualitative case study approach lies in the ability to generate deep contextual data to answer the ‘how’ and ‘why’ of complex social phenomena that may not be well understood [34].

### Ethics

The study was approved by the University of xx Human Research Ethics Committee (ethics ID 1544857.1) and the xx Human Research Ethics Committee (ethics ID H0015235). All participants provided written informed consent. Interpreters were available to assist participants to read participant information sheets and consent forms where required.

## Results

Participants in the ASPIRE project included immigrants and refugees who entered Australia via different immigration schemes, and who had quite varied pre-migration journeys. Some women had refugee backgrounds, having resettled through a humanitarian pathway, and were now permanent Australian residents or citizens. Other women had come to Australia on a range of temporary visas (including to work, for study, as a tourist, to seek asylum, as a spouse, or for a planned marriage), though many of these participants had subsequently become permanent residents or citizens. Some participants had migrated permanently as skilled workers. Some, but not all, women had experienced IPV and other forms of family violence prior to their arrival in Australia. The women were from a range of ages, nationalities, cultural groups, and religions. Participants came from South East Asia (n = 20, most commonly from Myanmar or the Philippines), the Indian subcontinent (n = 9), and various countries in Africa (n = 7), the Middle East (n = 4) and via the UK or Europe (n = 4). A small number of participants were second-generation immigrants born in either Australia or New Zealand (n = 4). Most were from backgrounds where English was not their first language. For detailed demographic information see Vaughan et al. [31].

Of the 46 participants interviewed, 13 described experiences that fit definitions of RC. These women were aged between 27 and 48 years, and were from Burkina Faso, India (2), Iraq (2), Mauritius (2), Myanmar, the Philippines (3), Sri Lanka and Vietnam. Where quotations are given below, pseudonyms are used to protect participant identity. Following the case study methodology as outlined by Baxter and Jack (2008) whereby particular “cases” are used to describe a phenomenon in context, we specifically examined immigrant and refugee women’s experiences of RC through data collected across multiple sites in metropolitan and regional Australia [31].

### Violence during pregnancy with intent to end the pregnancy

Violence during pregnancy was the most commonly experienced form of RC reported by ASPIRE participants. Celine, who had experienced IPV from when she was forced to marry at the age of 13, articulated that she was subject to specific violent acts that were explicitly intended to end her pregnancy.

When I was pregnant six months he want to kill it with a stick. He doesn’t want a baby. That day he want to “snap” my stomach. What I did, I put this knee straight like this to my stomach [demonstrates defensive posture] and my knee broke.

Women most often reported that their intimate partner was the perpetrator of physical violence aimed at causing a miscarriage. However, as was the case for some of the instances of forced abortion women reported, other family members enabled or encouraged this violence. Throughout her marriage, Katie had experienced emotional and verbal abuse from her mother-in-law. Her mother-in-law also disapproved of her pregnancy—a pregnancy that ended as a result of her husband’s physical violence.

I didn’t get my period and thought maybe stress. I went to the hospital and they said I was pregnant. I was happy even though it was not expected and we were trying to control it so that I didn’t fall pregnant, but it’s happened. I was happy. [My husband] came home two or three days he did not really speak to me. We started fighting.… I was standing and he said, “You want to lose the baby? I don’t want the baby. My mum said that we are too young to have a baby. How will we cope?” I said, “My parents will help me …I have milk. My parents will help me…” He said… “My mum doesn’t want the baby and I don’t want that baby.” He said, “You will lose this baby. I will make you lose this baby.” He pushed me on the bed so hard and I felt it in my tummy. I started crying but he didn’t come and hug me. The next morning I started bleeding.

### Forced pregnancy

Forced pregnancy is a well-recognised form of RC that undermines women’s reproductive autonomy. When children result from this coercion, this may lead to women feeling obliged to stay with their intimate partner. Even if they leave, this results in complicated and extended interactions with the father of their children. Julie described how her contraception was sabotaged, her movement restricted and that she was sexually assaulted in order to force a pregnancy.

The honest truth was that I never really wanted to get pregnant. I suppose to an extent it was sort of, I don’t know why I’m saying this, to an extent he forced himself on me. Locked me in a room.

Interviewer: Did the pregnancy come about in…

Julie: In a forceful way, yeah. Once I knew I was pregnant, given my religious beliefs and background, abortion was out of the question. God gave me this child, I’ve got to take responsibility…. My son’s father became very abusive once I was pregnant.

Interviewer: Did he exhibit abusive behaviours to you before pregnancy?

Julie: No… It was fairly good… I was working and he started to get a bit insecure. Then it was, you need to get pregnant, and it sort of started from locking me in my room, trying to get me pregnant. [The] idiot popped them all out, I used to take the pill and I used to hide it and he found it and popped them all out [of their packaging]. Fed them to the chooks. I couldn’t leave the room, like literally wetting myself in the corner of the room because I couldn’t get out. He locked me in there all day.

Interviewer: Did he want to get you pregnant because he didn’t want you working? He wanted to keep you at home?

Julie: He wanted to keep me there. He wanted to keep me at home. If I was pregnant I wasn’t going nowhere. Who’s going to want her with a child? She’ll never leave.

### Forced abortion

Several women recounted stories of being forced to have an abortion by their partner, or other/multiple members of their partner’s family. These cases highlight how violence and intimidation were used to reduce women’s agency regarding their reproductive decisions. In Bharti’s case presented below, extended family members (her husband’s family) called health services to arrange appointments in her name, and those who accompanied her to the appointments liaised with health professionals on her behalf, despite her wishes not to complete the procedure.

Bharti: I got pregnant in July…then he forced me to have an abortion… My husband rang [partner’s mother] to help me to take me to take to the hospital, but he didn’t want to go with me…He was not with me at the time. [Partner’s mother] did everything. She took me to the hospital and she stayed with me at my place for two or three days. The next day he just went to the gym. He doesn’t care.

Another participant, Mannat, experienced sustained and severe violence from her husband and his family. When she became pregnant, her sister-in-law joined forces with her husband in his efforts to end the pregnancy and made calls to health professionals pretending that she was Mannat.

My sister-in-law gave me the test and said if I was pregnant, I had to have an abortion. I told her I’m not going to abort to this child; it was my child. I told her I couldn’t kill anyone, so she started punching the wall…She was the dominant person, so I was afraid that without her permission, she would stop the baby. My husband didn’t listen to me and I cried the whole night…

So, she [sister-in-law] booked an abortion and made an appointment… I was crying and saying I could go back [to India] if they couldn’t afford the baby. I’d go back to my country and stay with my parents, but I couldn’t kill my baby. He didn’t listen.

In addition to being a case of forced abortion, Mannat’s experience highlights the ways in which perpetrators of violence may control women’s access to and information about health services. In Mannat’s case, this included through manipulating health workers’ assumption that she did not speak English (though she did very well).

For the confidentiality, they have to take a signature and they took me in the room. I said I wasn’t going to sign but my husband said I didn’t know English and he understood a bit, so he accompanied me to sign it. I was too scared [to tell them I did speak English] because the choking, hitting and abusing was too much.

Mannat’s case also illustrates the ways that providers of reproductive health care may facilitate the perpetration of RC by allowing partners or family members to mediate communication in health settings and failing to ensure informed consent to health procedures. Conversely, Mannat’s case also illustrates the potential role of health professionals in identifying IPV and, in particular, RC. For Mannat, like many other women interviewed during the ASPIRE project, health care settings were a lost opportunity to disclose what she was experiencing and access help.

## Discussion

Whilst RC and abuse has been defined in the literature as a form of IPV for around a decade [9], only a small body of literature on its prevalence and mechanisms exist [11]. Whilst there is some evidence that RC is experienced more by young women [16, 17], and generally co-occurs with other forms of violence [2, 13], a comprehensive picture of how RC occurs within particular populations of women is not available. This is the first study that specifically explores how immigrant and refugee women have experienced RC, and the case studies presented in this paper highlight the differing contexts and range of perpetrators involved in RC as experienced by this population. The ASPIRE project [31] provided a unique opportunity to add insight into how immigrant and refugee women living in Australia had experienced RC.

Whilst the majority of women in the ASPIRE project reported that their husband/male partner was the main perpetrator of violence against them, in some cases other males and/or members of the extended family (e.g., mothers-in-law or sisters in-law) were involved in multi-perpetrator violence [31]. This also appeared to be a feature of descriptions of RC in this analysis, as women who described forced abortion often reported that multiple family members co-ordinated the procedure and were involved in victimisation of the participant. Similarly, women who experienced violence during pregnancy with intent to end the pregnancy mentioned the influence of other family members on their husband/ partner’s attitude towards the pregnancy. An Australian study [35] notes that some health professionals are aware of potential gender-based violence (control) around contraception use for some migrant/refugee women. This study suggests however, that service providers may be unaware of additional forms of RC, whereby victims from immigrant and refugee backgrounds may not present as a “typical” case of IPV, and extended family (including women) may be complicit in coercing women’s health care decisions.

Whilst definitions of what constitutes RC between a woman and her intimate partner have been described in previous studies, literature exploring how health and human services, and victims themselves understand RC is still emerging [1]. It is widely acknowledged that RC intersects and coexists with other forms of intimate partner violence, particularly sexual violence. In this study, all women were initially interviewed because they had experienced other forms of family violence, and RC emerged in interviews as additional to these. Tarzia and colleagues [1] notes that a key issue with defining and researching RC is clarifying how it relates to both sexual violence and/ or intimate partner violence, and a strong theme that delineates RC from these other forms of violence is perpetrator intent. This could involve a perpetrator using other forms of violence with the intention to control a particular reproductive outcome (e.g., pregnancy, miscarriage, termination), or reproductive outcomes (e.g., pregnancy) occurring as a consequence of other forms of IPV. Whilst RC may be only one aspect of IPV experienced by a woman, it is useful to identify RC discretely as it raises specific issues for how to appropriately respond to the abuse and connect women to services [36]. Legal definitions of sexual violence are not always well understood by women who experience it, and women of immigrant and refugee background may be less aware of specific Australian laws surrounding domestic and family violence [1, 21, 26] Women’s reproductive health outcomes are inherently linked to sexual experiences, and in this study, RC did coexist with sexual violence for a number of women, especially (and unsurprisingly) in cases of forced pregnancy. In this study, women also reported RC as co-occurring with other forms of coercion including physical violence (in cases of violence with the intention to cause miscarriage) and extreme controlling behaviours (e.g., being locked in rooms, controlling employment decisions, threats about visa status being revoked if women left).

Immigrant and refugee women in this study described experiences of multiple perpetrators controlling their use of health care by either denying access to care, using intimidation to influence health decisions, and/or orchestrating women to have procedures against their wishes. This experience was described across different types of RC and provides insight into additional forms of violence that immigrant and refugee women may face in relation to RC and the ways that structural inequality within the health system contributes to immigrant and refugee women’s experience of RC. It is well documented that there is a number of possible health implications associated with RC (increased risk of STIs, unwanted pregnancy). There is also evidence that women may use health services as an opportunity to seek help for IPV, and that women experiencing RC are more likely to seek reproductive health care than women who do not [37–39]. However, the structural inequality that is embedded within the Australian health system means that immigrant and refugee women face additional barriers to accessing reproductive health care compared to the general Australian population (e.g., language barriers) [31], and nascent research has noted that migrant or culturally and linguistically diverse women may be particularly vulnerable to RC [11, 40]. The findings of this study support this, showing how perpetrators are able to manipulate inequitable health service gaps to undermine immigrant and refugee women’s reproductive choices. The findings also show a failure of health professionals to communicate appropriately and directly with immigrant and refugee women to support them to advocate for their own health decisions.

### Limitations of our research

As the findings presented in this paper are the result of a case-study analysis of available qualitative data, some limitations were inherent in this research. Whilst the original interview questions elicited responses about a broad range of experiences of violence, specific questions using standard definitions of RC were not included. Rather, discussion of RC commonly emerged during interviews in relation to other violent experiences or when participants described their reproductive history. Hence it is likely that other participants in the study had suffered RC but this was not disclosed. Most of the women who participated were referred to the study by family violence, health or settlement services, which means the experiences recounted may not reflect those of women with no connection to service providers when dealing with family violence. Also, interviews were conducted with women who had come to Australia through a wide range of migration journeys (the humanitarian, family and skilled streams of the migration program, a range of temporary visas (including partner), women seeking asylum and second-generation migrants). Recruitment may not have represented women who face particular forms of discrimination e.g. women with disabilities or those who face violence in same-sex relationships [31].

## Conclusions

The findings of this study provide some of the first descriptions of RC as experienced by immigrant and refugee women from the ASPIRE study, and as such, provide guidance about further research that is necessary to understand and address RC in this population. More information is needed about the interactions between health services and immigrant and refugee women experiencing RC, and how vulnerability to multi-perpetrator violence affects health service access. In particular, knowledge about how multi-perpetrator RC can affect consent processes for women who already face systemic barriers to health care requires attention. Further research is required to address knowledge gaps about how to conduct appropriate prevention and advocacy work about RC in refugee and migrant communities, and what training is needed for professionals in the family violence sector, women’s health services, women’s organisations, multicultural and ethno-specific services. Knowledge about how health systems, migration policy and systems and other services can ensure their practices and policies do not inadvertently facilitate RC is also necessary.
